# Mesorectal excision with lateral lymph node dissection for mid-low rectal cancer with lateral lymph node metastasis: efficacy and prognostic analysis

**DOI:** 10.1186/s12957-022-02574-6

**Published:** 2022-03-29

**Authors:** Sicheng Zhou, Yujuan Jiang, Jianwei Liang, Qian Liu

**Affiliations:** Department of Colorectal Surgery, National Cancer Center/National Clinical Research Center for Cancer/Cancer Hospital, Chinese Academy of Medical Sciences and Peking Union Medical College, Beijing, 100021 China

**Keywords:** Mesorectal excision, Lateral pelvic lymph node dissection, Prognosis

## Abstract

**Background:**

The aim of this study was to evaluate the efficacy of lateral pelvic lymph node (LPN) dissection (LPND) for rectal cancer patients with LPN metastasis (LPNM) and investigate the impact of LPNM on prognosis.

**Methods:**

One hundred twenty-five matched pairs were selected and divided into the total mesorectal excision (TME) group and TME + LPND group for evaluation after propensity matching.

**Results:**

No significant difference was observed in the 3-year local recurrence rate between the TME group and the TME + LPND group (10.7% vs 8.8%, *P* = 0.817); however, the rate of distant metastasis after TME + LPND was significantly higher (15.2% vs 7.2%, *P* = 0.044). When the mesorectal LN and LPN groups were subdivided, 3-year RFS was not significantly different between the internal LPN and N2 groups (57.1% vs. 55.3%, *P* = 0.613). There was no significant difference in RFS between the external group and the stage IV group (49.1% vs. 22.5%, *P* = 0.302), but RFS in the former group was significantly worse than that in the N2 group (49.1% vs. 55.3%, *P* = 0.044).

**Conclusion:**

Although patients with suspected LPNM can achieve satisfactory local control after TME + LPND, systemic metastases are more likely to develop after surgery. Patients limited to internal iliac and obturator LN metastasis appear to achieve a survival benefit from LPND and can be regarded as regional LN metastasis. However, patients with LPNM in the external and common iliac LN metastasis have a poor prognosis that is significantly worse than that of N2 and slightly better than that of stage IV, and LPND should be carefully selected.

## Introduction

Mesorectal excision (ME) or total mesorectal excision (TME) is currently an internationally recognized surgical principle for middle-low rectal cancer. However, approximately 7–23% of patients with stage II or III middle-low rectal cancer occasionally develop metastases to the lateral pelvic lymph nodes (LPNs), which are outside the scope of surgical dissection of the TME or ME and are associated with poor prognosis and higher local recurrence rates [1–5]. In Japan, LPNs are regarded as regional lymph nodes, which are considered within the scope of the N3 stage. The JCOG (Japanese Clinical Oncology Group) 0212 trial demonstrated the safety and oncology efficacy of LPN dissection (LPND) [1, 2]. The results of this trial revealed that TME + LPND has satisfactory perioperative outcomes, with similar postoperative morbidity and mortality as TME alone. Furthermore, TME with LPND resulted in a lower local recurrence, especially in the lateral pelvis, compared to ME alone. Therefore, the JSCCR guidelines suggested that TME + LPND should be performed routinely for patients with stage II/III middle-low rectal cancer [6].

In contrast, several studies, also from Japan, suggested that the overall benefit related to local control and survival of LPND is not promising in patients with LPN metastasis (LPNM) [4, 7–11]. We suggest that there are two reasons for the difference in the results reported above. First, only surgeons who specialized in TME + LPND participated in the JCOG trial. Therefore, this technically demanding procedure may yield different survival outcomes in different institutions. More importantly, the JCOG trial excluded almost 20% of patients with clinically suspected LPNM diagnosed by preoperative imaging from the study, which is not in line with the real situation in clinical practice. Therefore, it is necessary to clarify the effectiveness of TME + LPND with regard to increasing local control and prolonging survival for patients with LPNM in different regions and institutions. In addition, we found that patients with LPNM located in the area of the internal iliac tended to achieve a better prognosis. The present study aimed to evaluate the efficacy of LPND for middle-low rectal cancer patients with LPNM and investigate the impact of LPNM on survival outcomes.

## Patients and methods

### Patients

**Fig. 1:**
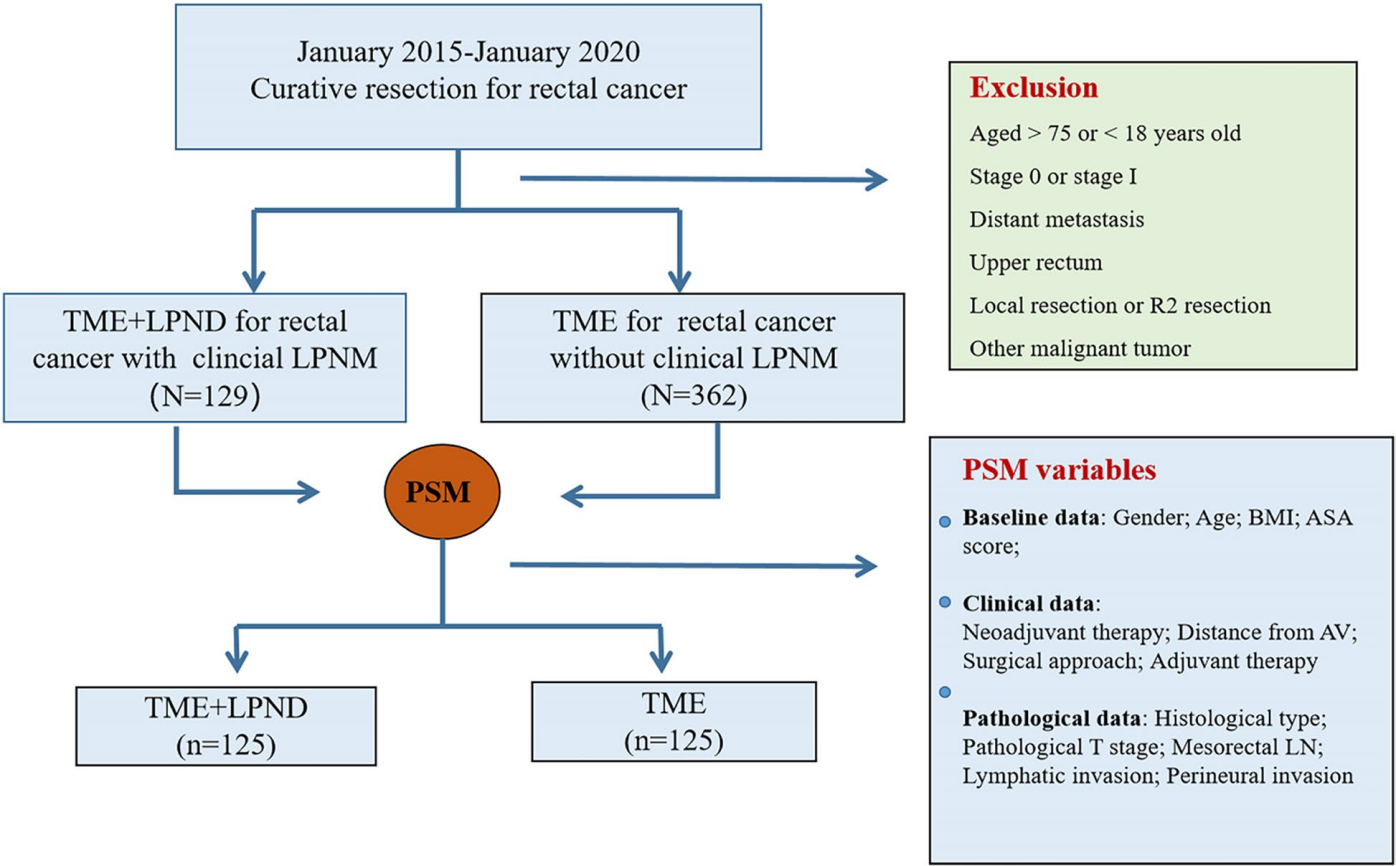
Group flow chart

From January 2015 and January 2020, we reviewed the records of 129 middle-low rectal cancer patients with clinical LPNM who underwent TME + LPND at the National Cancer Center/National Clinical Research Center for Cancer/Cancer Hospital, Chinese Academy of Medical Sciences and Peking Union Medical College. Data of 362 consecutive patients with middle-low rectal cancer who underwent curative surgery with TME during the same period were also collected and reviewed. The inclusion criteria were as follows: (1) 18–75 years old, (2) lower margin of rectal tumour below the peritoneal reflection, (3) clinical diagnosis of stage II–III, and (4) pathological diagnosis of adenocarcinoma. Patients who underwent transanal local excision or had a history of other malignant tumours were excluded from the study. All enrolled patients were divided into the TME + LPND group and the TME group according to the surgical methods and included in the propensity score matching (PSM) process, and 125 matched pairs were eventually selected (Fig. 1). The study protocol was approved by the Ethics Committee of the Cancer Hospital, Chinese Academy of Medical Sciences (NCC 2017-YZ-026, October 17, 2017), and written informed consent was obtained from all enrolled patients.

Preoperative investigations for all patients included laboratory examination, endoscopy, pelvic magnetic resonance imaging (MRI), and computed tomography (CT) of the abdomen. Clinical stage and LPN status were evaluated according two imaging radiologists who specialized in colorectal cancer. LPND was performed for stage II–III patients with suspected clinical LPN metastasis, and LPN metastasis can be diagnosed by meeting one or more of the following diagnostic criteria: (1) ≥ 0.5 cm in short diameter before treatment, (2) inhomogeneous or intense enhancement, and (3) irregular shape and rough edges. Treatment strategies for each patient, such as the choice of surgical approach (open or laparoscopic) and whether neoadjuvant therapy was performed, were determined by patients’ wishes and multidisciplinary team meetings that incorporated radiologists and medical and surgical oncologists. All procedures were performed by surgeons with more than 20 years of experience in colorectal surgery. At our institution, there is no standard indication for LPND. The surgical method was ascertained at the discretion of the surgeon with full consideration of the patient’s characteristics, pathological results, and preoperative examination. The American Joint Committee on Cancer (AJCC) staging Manual (8th edition) was used for tumour staging [12]. According to the JSCCR classification, LPN areas were divided into four regions: obturator, external iliac, internal iliac, and common iliac [13]. Patients were divided into three groups according to the status of lymph node (LN) metastasis: (1) patients without any LN metastasis were classified into the N0 group, (2) patients with mesenteric LN metastasis without LPNM were classified into the mesorectal LN group, and (3) patients with LPNM, regardless of mesenteric LN status, were divided into the LPN group.

Patients were followed-up through outpatient clinic or telephone after operation, including survival, causes of death, and the follow-up period. The follow-up deadline was the recurrence date or February 1, 2021, whichever came first. Serum tumour markers, abdominal CT examinations, and colonoscopy were performed every 3 months for the first 3 years and every 6 months for the next 2 years. The long-term endpoint of this study was 3-year local recurrence rate and 3-year recurrence-free survival (RFS), and the data were collected based on this follow-up survey.

### Statistical analysis

Patients in the TME group were matched in a 1:1 ratio to those in the TME + LPND group through PSM based on the following factors: age, sex, BMI, ASA score, neoadjuvant therapy, distance from AV, histological type, pathological T stage, mesorectal LN, surgical approach, lymphatic invasion, perineural invasion, and adjuvant therapy.

Clinical and pathological data are expressed as frequencies and percentages or means ± standard deviations. The differences in variables between the two groups were measured by the *χ*^2^ test or Fisher’s exact test. The RFS curves were calculated by the Kaplan-Meier method and compared by the log-rank test. The predictors determined to have a *P* value < 0.05 in univariate analysis were subsequently tested by multivariate analysis through a Cox proportional hazards model, and hazard ratios (HRs) with a 95% confidence interval (CI) were reported for each variable. A *P* value less than 0.05 was considered statistically significant. Data statistics and analysis were performed using SPSS for Windows version 20.0 (SPSS, Chicago, Illinois, USA).

## Results

### Patient characteristics

The baseline data, clinical parameters and pathological characteristics are presented in Table 1. A total of 125 marched pairs were selected through propensity scoring and enrolled in the study. After matching, the TME group and TME + LPND group were well balanced in terms of age, sex, BMI, ASA score, preoperative CEA level, preoperative CA19-9 level, preoperative treatment, distance from AV, histological type, pathological T stage, mesorectal LN, status of LN metastasis, LPLNs harvested, mesorectal LNs harvested, lymphatic invasion, perineural invasion, and adjuvant therapy.

**Table 1:** Clinical and pathological characteristics of rectal cancer patients who underwent TME with or without LPND before and after matching

Variables	Original cohort			Matched cohort		
	TME (*n* = 362)	TME + LPND (*n* = 129)	*P*	TME (*n* = 125)	TME + LPND (*n* = 125)	*P*
Gender			0.493			0.798
Male	195 (53.9)	74 (57.3)		71 (56.8)	73 (58.4)	
Female	167 (44.1)	55 (42.7)		54 (43.2)	52 (41.6)	
Age (years)			0.869			0.899
< 60	199 (55.0)	72 (55.8)		68 (54.4)	69 (55.2)	
≥ 60	163 (45.0)	57 (44.2)		57 (45.6)	56 (44.8)	
BMI (kg/m^2^)	24.0 ± 3.1	24.9 ± 3.2	0.081	24.5 ± 3.1	25.0 ± 3.1	0.226
ASA score			0.784			0.625
I–II	337 (93.1)	121 (93.8)		115 (92.0)	117 (91.4)	
III	25 (6.9)	8 (6.2)		10 (8.0)	8 (8.6)	
Neoadjuvant therapy			0.284			0.613
Yes	157 (43.4)	63 (48.8)		66 (52.8)	62 (49.6)	
No	205 (56.6)	66 (51.2)		59 (47.2)	63 (50.4)	
Distance from anal verge (cm)	5.5 ± 1.4	5.1 ± 1.4	0.339	5.4 ± 1.5	5.1 ± 1.4	0.452
Histological type			0.025			0.172
Well/moderate	290 (80.1)	91 (70.5)		81 (64.8)	91 (72.8)	
Poor/mucinous/signet	72 (19.9)	38 (29.5)		44 (35.2)	34 (27.2)	
Pathological T stage			0.302			0.506
T1–T2	71 (19.6)	20 (15.5)		24 (19.2)	20 (16.0)	
T3–T4	291 (80.4)	109 (84.5)		101 (80.8)	105 (84)	
Mesorectal LN			< 0.001			0.855
N0	151 (41.7)	31 (24.0)		25 (20.0)	28 (22.4)	
N1	149 (41.2)	60 (46.5)		63 (50.4)	59 (47.2)	
N2	62 (17.1)	38 (30.4)		37 (29.6)	38 (30.4)	
LPN metastasis						–
Presence	–	26 (20.2)		–	25 (20.0)	
Absence	–	103 (79.8)		–	100 (80.0)	
Surgical approach			0.402			0.684
Open	21 (5.8)	5 (3.9)		2 (1.6)	4 (3.2)	
Laparoscopic	341 (94.2)	124 (96.1)		123 (98.4)	121 (96.8)	
Lymphatic invasion	78 (21.5)	37 (28.7)	0.100	35 (28.0)	34 (27.2)	0.887
Perineural invasion	92 (25.4)	47 (36.4)	0.017	48 (38.4)	45 (36.0)	0.665
Adjuvant therapy	126 (34.8)	82 (63.6)	< 0.001	83 (66.4)	80 (64.0)	0.690

### Operative detail and postoperative complications

The perioperative outcomes of the patients are summarized in Table 2. Low anterior resection and abdominoperineal resection were performed in 121 (48.4%) and 123 patients (49.2%), respectively. Thirty (24.0%) patients in the TME + LPND group underwent bilateral LPND. TME with LPND required a significantly longer operation time (356.1 vs. 244.8 min, *P* < 0.001) and resulted in a similar estimated blood loss (78.7 vs. 64.1 ml, *P* = 0.202) than TME alone. In terms of postoperative complications, there was no significant difference between the two groups (16.0 vs. 12.0, *P* = 0.362), and each complication was similar. The time to first flatus (3.1 vs. 3.3 days, *P* = 0.552) and the postoperative hospital stay (8.5 vs. 8.8 days, *P* = 0.630) were not significantly different between the groups. All patients recovered from surgery and were discharged from the hospital, and no deaths were observed during the perioperative period in either group.

**Table 2:** Perioperative detail of rectal cancer patients who underwent TME with or without LPND after matching

Variables	TME (*n* = 125)	TME + LPND (*n* = 125)	*P*
Types of operation			0.623
Low anterior resection	63 (50.4)	58 (46.4)	
Abdominoperineal resection	60 (48.0)	63 (50.4)	
Hartmann procedure	2 (1.6)	4 (3.2)	
LPND			–
Unilateral dissection	–	95 (76.0)	
Bilateral dissection	–	30 (24.0)	
Operative time (min)	244.8 ± 60.3	356.1 ± 76.5	< 0.001
Estimated blood loss (ml)	64.1 ± 97.2	78.7 ± 81.7	0.202
Postoperative complications	15 (12.0)	20 (16.0)	0.362
Postoperative bleeding	3 (2.4)	2 (1.6)	
Ileus	1 (0.8)	3 (2.4)	
Anastomosis leakage	4 (3.2)	2 (1.6)	
Pelvic cavity abscess	5 (4.0)	3 (2.4)	
Pneumonia	3 (2.4)	6 (4.8)	
Wound infection	2 (1.6)	5 (4.0)	
Urinary retention	1 (0.8)	2 (1.6)	
Rectovaginal fistula	1 (0.8)	0 (0)	
Time to first flatus (days)	3.3 ± 1.2	3.1 ± 1.4	0.552
Postoperative hospital stay (days)	8.8 ± 5.8	8.5 ± 5.8	0.630
Re-operation	3 (2.4)	1 (0.8)	0.622
Mortality	0 (0)	0 (0)	–

### Local recurrence and distant metastasis

**Fig. 2:**
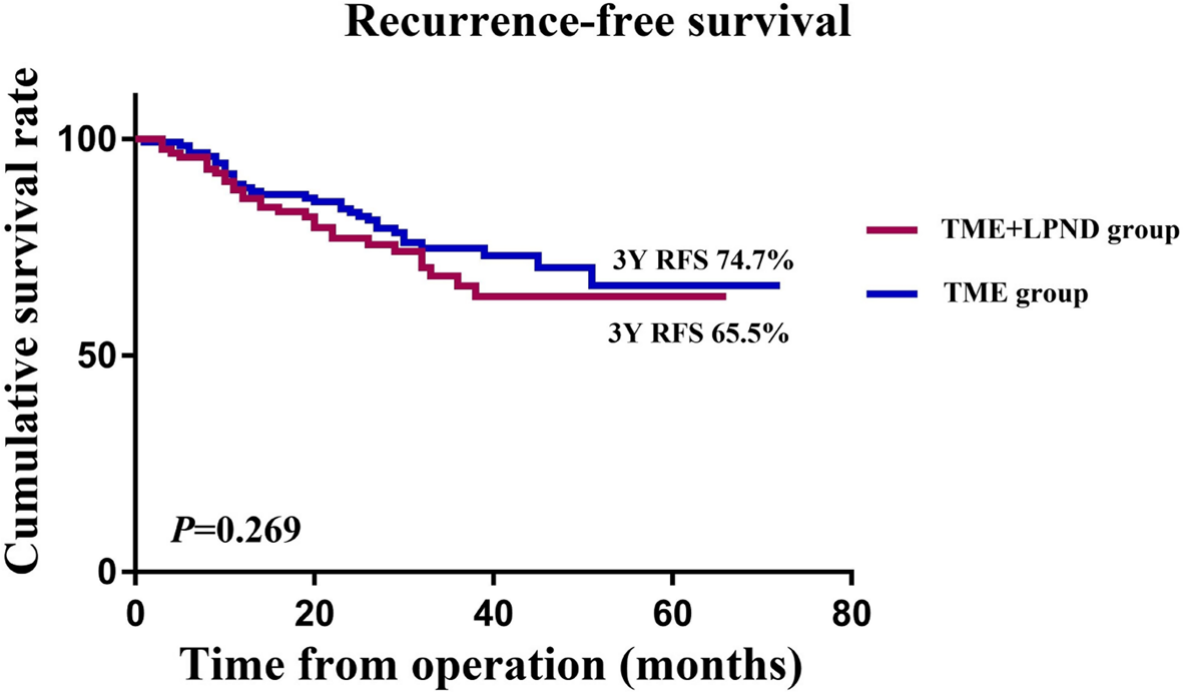
Recurrence-survival rate of rectal patients in the TME + LPND group (*n* = 125) and TME group (*n* = 125)

**Fig. 3:**
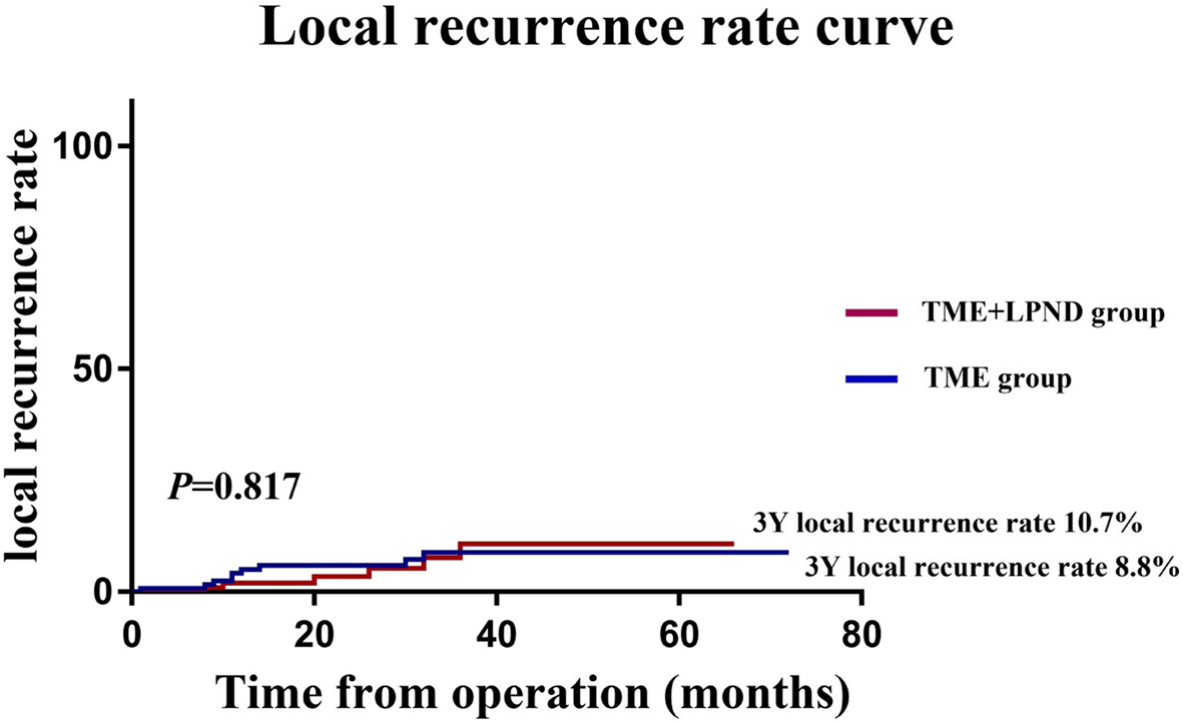
Local recurrence rate of rectal patients in the TME + LPND group (*n* = 125) and TME group (*n* = 125)

The mean follow-up period of the whole group was 44.0 (range, 3–72) months. Of all 250 patients undergoing curative resection for rectal cancer, 33 (13.2%) had a recurrence of cancer up to 3 years after surgery, including 11 (4.4%) local recurrence cases and 28 (11.2%) distant metastasis cases. The rates of overall recurrence (16.8% vs. 9.6% *P* = 0.093) and local recurrence (4.8% vs. 4.0%, *P* = 0.758) did not differ between the TME + LPND group and the TME group (Table 3). However, the distant metastases rate was significantly higher in the TME + LPND group than in the TME group (15.2% vs 7.2%, *P* = 0.044). In addition, the 3-year RFS rate (65.5% vs. 74.7%, *P* = 0.269) and 3-years local recurrence rate (10.7% vs 8.8%, *P* = 0.817) was similar in both groups (Fig. 2, Fig. 3).

**Table 3:** Postoperative recurrence of 250 rectal cancer patients who underwent TME with or without LPND up to 3 years after surgery in matching cohort

	TME (*n* = 125)	TME + LPND (*n* = 125)	*P*
Overall recurrence (%)	12 (9.6)	21 (16.8)	0.093
Local recurrence	5 (4.0)	6 (4.8)	0.758
Distant metastasis	9 (7.2)	19 (15.2)	0.044
Liver metastasis	5 (4.0)	11 (8.8)	
Lung metastasis	4 (3.1)	8 (6.3)	
Bone metastasis	4 (3.1)	5 (4.0)	
Peritoneal metastasis	0 (0)	1 (0.8)	
Brain metastasis	1 (0.8)	0 (0)	
Others	1 (0.8)	2 (1.6)

### Prognostic factors

To determine prognostic factors for RFS of patients with middle-low rectal cancer, univariate and multivariate regression analyses were performed (Table 4). In univariate analysis, preoperative CEA level > 5 ng/ml, perineural invasion, and status of LN metastasis significantly affected RFS (*P* < 0.05). The prognostic factors determined to have a *P* value < 0.05 in univariate analysis were subsequently tested by multivariate analysis through a Cox proportional hazards model and the results demonstrated that independent prognostic factors associated with RFS were the status of LN metastasis.

**Table 4:** Univariate and multivariate analyses for recurrence-free survival of the 250 rectal patients who underwent TME with or without LPND in matching cohort

Variables	Recurrence-free survival			
	Univariate analysis		Multivariate analysis	
	HR (95% CI)	*P*	HR (95% CI)	*P*
Gender: male	1.36 (0.81–2.27)	0.244		
Age: ≥ 60 years	1.08 (0.66–1.78)	0.751		
Preoperative CEA level: > 5 ng/ml	1.70 (1.02–2.83)	0.043	1.55 (0.92–2.61)	0.097
Histology: other	1.44 (0.87–2.39)	0.155		
Lymphatic invasion: yes	1.55 (0.92–2.62)	0.103		
Perineural invasion: yes	1.75 (1.06–2.86)	0.027	1.53 (0.85–2.76)	0.157
T stage: T3–4	1.63 (0.87–3.06)	0.130		
Status of LN metastasis				
N0	Reference		Reference	
Mesorectal-LN	1.82 (1.01–3.26)	0.045	1.95 (1.10–3.60)	0.044
LPN	4.06 (1.79–9.20)	0.001	3.03 (1.23–7.47)	0.016
LPND: yes	1.32 (0.80–2.17)	0.269		
Adjuvant chemotherapy: yes	0.94 (0.56–1.58)	0.812		
Postoperative complications: yes	1.09 (0.56–2.15)	0.799		

**Fig. 4:**
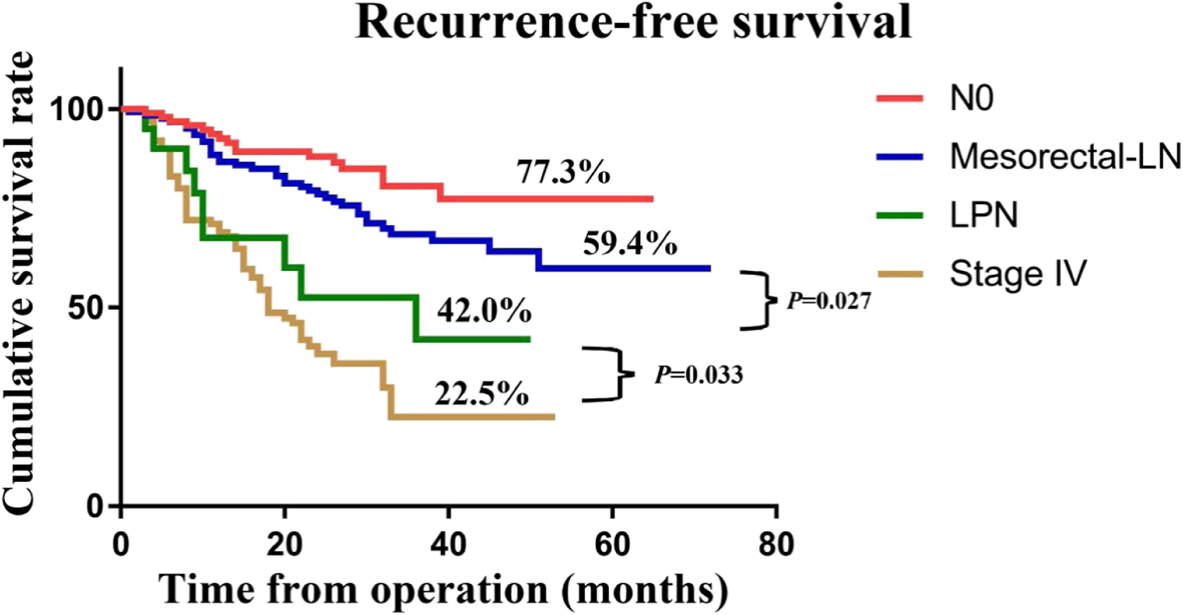
Recurrence-free survival for 250 patient who underwent TME with or without LPND and 100 patients with stage IV who underwent curative resection

Overall, 51 (20.4%), 174 (69.6%), and 25 (10.0%) patients were classified into the N0, mesorectal-LN, and LPN groups, respectively. To investigate the prognostic difference between LPNM, regional mesorectal LN metastasis, and distant metastasis, the survival of 100 patients with stage IV rectal cancer who underwent curative resection from June 2017 to June 2019 was retrospectively collected and analysed. The 3-year RFS of patients with LPNM was significantly better than that of stage IV patients (52.3% vs. 22.5%, *P* = 0.033) and significantly worse than that of patients with mesorectal LN metastasis (52.3% vs. 65.2%, *P* = 0.027) (Fig. 4).

**Fig. 5:**
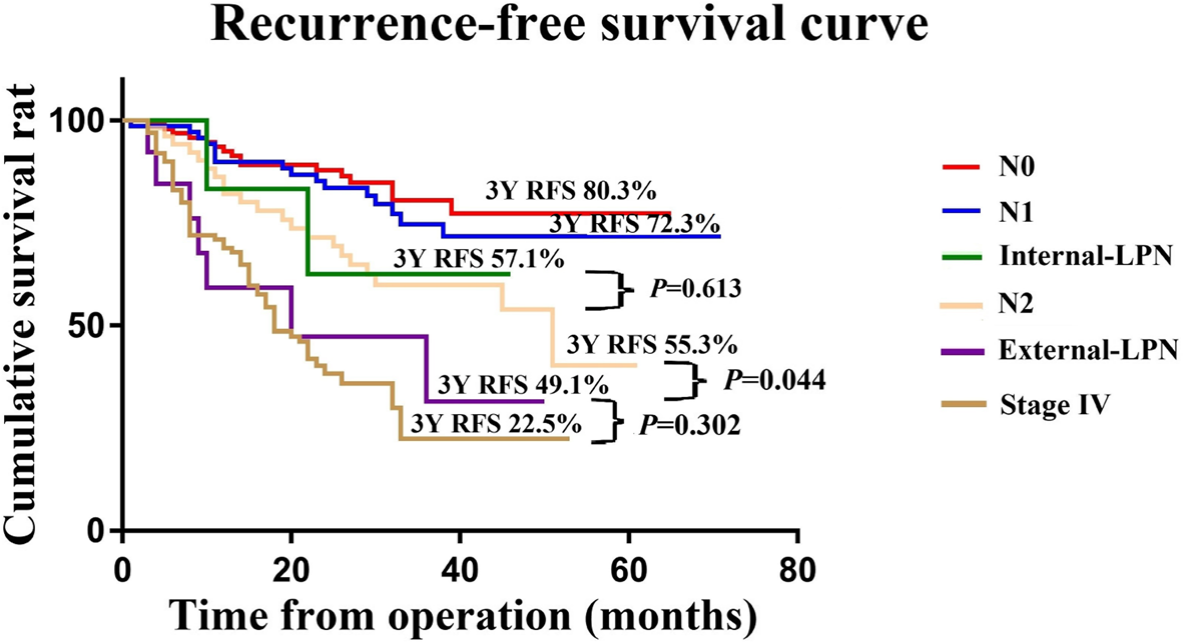
The recurrence-free survival for 250 patients who underwent TME with or without LPND after subgroup analysis and 100 patients with stage IV who underwent radical resection

To further clarify the influence of the location of LPNM on prognosis, we subdivided the mesorectal LN group into two groups according to the AJCC tumour staging system (N1: 1-3 regional LN metastasis; N2: > 3 regional LN metastasis). The LPN group was also subdivided into two categories (internal LPN: internal iliac and obturator LN metastasis; external LPN: external and common iliac LN metastasis). The 3-year RFS rates of the N0, N1, internal LPN, N2, external LPN, and stage IV groups were 80.3%, 72.3%, 57.1%, 55.3%, 49.%, and 22.5%, respectively (Fig. 5). RFS was not significantly different between the internal-LPN and N2 groups (*P* = 0.613) or between the external-LPN and stage IV groups (*P* = 0.302). Notably, the RFS of the external-LPN group was significantly worse than that of the N2 group (*P* = 0.044).

## Discussion

In the present study, among the 125 patients who underwent TME + LPND, the pathologically confirmed rate of LPNM was 20.0% (25/125), which is consistent with reporting rates varying from 8.6 to 18.6% in the previous literature [8, 14, 15]. To minimize the impact of selection bias on the results, PSM was carried out to balance baseline data and clinicopathological characteristics between the two groups, and all patients included in this study were operated on by surgeons at the National Cancer Center with more than 20 years of experience in colorectal surgery. Therefore, this study is more in line with the real situation in clinical practice and better provides practice-based evidence.

The long-term survival outcomes determine the value and effectiveness of LPND for rectal cancer. A large multi-centre study conducted by Kobayashi et al. analysed 1272 low rectal cancer patients and they concluded that LPND may be beneficial for specific patients [4]. However, it has been previously reported that LPND does not significantly improve local control and survival [7–11]. A phase III randomized controlled study of 445 patients with stage II/III rectal cancer conducted by Oki and his colleagues showed that LPND had no impact on RFS (HR = 0.941, 95% CI: 0.696–1.271, *P* = 0.69) or overall survival (HR = 0.858, 95% CI: 0.601–1.224, *P* = 0.39) in all patients [10]. Our study demonstrated that there was no significant difference in the 3-year local recurrence rate between the TME group and the LPND group (10.7% vs 8.8%, *P* = 0.817); however, the rate of distant metastasis after TME + LPND was significantly higher than that in the TME group (15.2% vs 7.2%, *P* = 0.044). It suggests that TME + LPND can achieve satisfactory control effects in patients with clinical suspicion of LPNM; however, there is a potential for micro-metastasis in such patients, so intensive systemic chemotherapy is a reliable way to reduce distant metastases.

It is well known that LPNM is associated with local recurrence and poor long-term survival. A retrospective study involving 149 patients conducted by Sato et al. proved that patients with LPNM were more likely to relapse, and LPNM was an adverse prognostic factor for patients with rectal carcinoma below the peritoneal reflection [7]. The present study also revealed that mesorectal LN metastasis (HR: 1.95; 95% CI, 1.10–3.60; *P* = 0.044) and LPNM (HR: 3.03; 95% CI, 1.23–7.47; *P* = 0.016) were independent poor predictive factors affecting RFS, which agrees with the data previously reported in the literature [7, 10]. This may be due to the fact that patients with LPNM are mostly advanced stage, which is often complicated with regional lymph node metastasis and distant micro-metastasis. Therefore, this further demonstrates the importance and significance of exploring the indications for LPND.

To further explore the prognostic significance of different LPNM locations, we investigated and compared the prognostic differences between LPNM, regional mesorectal LN metastasis and distant metastasis. The results demonstrated that the RFS rate of patients with LPNM was significantly better than that of stage IV patients (*P* = 0.033) and significantly worse than that of patients with mesorectal LN metastasis (*P* = 0.027). Previous literature has reported that LPND may provide survival benefits for patients with LPNM in the internal iliac vessel region or the obturator region [5, 7, 16, 17]. A Japanese nationwide multi-institutional study enrolled 11,567 patients with stage I-III rectal cancer and revealed that both the overall survival (OS) and cancer-specific survival (CCS) of patients with internal LPN (*P* = 0.9585 for OS and 0.5742 for CSS) and external LPN metastases (*P* = 0.3342 for OS and 0.4347 for CSS) were similar to those of patients with N2a and N2b stages, respectively. Furthermore, both the OS and CSS of the patients with external LPN metastasis were significantly better than those with stage IV metastasis. (*P* = 0.024 for OS and 0.011 for CSS) [5].

In the present study, we further subdivided each group and found that RFS was not significantly different between the internal-LPN and N2 groups (*P* = 0.613). However, in contrast to the literature reported above, our results showed that the RFS of patients with external LPN metastasis was not significantly different from that of patients with stage IV rectal cancer (*P* = 0.302) and was significantly lower than that of patients with stage N2 rectal cancer (*P* = 0.044). We suggested that the 5-year rate of RFS in patients with external LPN metastasis is approximately 10% higher than that in stage IV rectal patients (31.6% vs. 22.5%), possibly due to the small sample size, which could not achieve a significant difference. We did not further subdivide patients by N2 stage, which made it impossible to describe in detail the difference in prognosis between LPNM and mesorectal LN metastasis. It is noteworthy that most of above Japanese evidence based on the results of patients without neoadjuvant therapy [1, 2, 5]; however, in this study, some patients receiving neoadjuvant therapy were included, which could lead to the complete elimination of LPNM, thus affecting the prognostic analysis.

Several limitations of the present study should be clarified and considered. The first potential limitation involves the selection bias caused by the retrospective nature. Theoretically, patients selected for LPND may have advanced disease and a higher rate of LPNM. However, we performed PSM to balance the cohort as far as practicable. Second, the sample size of this study was small, and only 250 patients with rectal cancer were included for discussion and analysis. Thirdly, neoadjuvant therapy could completely eliminate LPNM, and these patients are classified as negative LPNs, thus affecting the outcome of the prognostic analysis. Therefore, a multi-centre randomized controlled trial is needed to further verify our conclusions.

## Conclusion

Although patients with suspected LPNM can achieve satisfactory local control after TME + LPND, systemic metastases are more likely to develop after surgery. Patients limited to internal iliac and obturator LN metastasis appear to achieve a survival benefit from LPND and can be regarded as regional LN metastasis. However, patients with LPNM in the external and common iliac LN metastasis have a poor prognosis that is significantly worse than that of N2 and slightly better than that of stage IV, and LPND should be carefully selected.

## Data Availability

The datasets generated and/or analysed during the current study are not publicly available due to the data is confidential patient data but are available from the corresponding author on reasonable request.

## Abbreviations

LPND: Lateral pelvic lymph node dissection
LPNM: Lateral pelvic lymph node metastasis
TME: Total mesorectal excision
LN: Lymph node
RFS: Recurrence-free survival
ME: Mesorectal excision
MRI: Pelvic magnetic resonance imaging
CT: Computed tomography
AJCC: American Joint Committee on Cancer
HRs: Hazard ratios
CI: Confidence interval
